# Heterologous and Homologous Expression of Proteins from Haloarchaea: Denitrification as Case of Study

**DOI:** 10.3390/ijms21010082

**Published:** 2019-12-20

**Authors:** Rosa María Martínez-Espinosa

**Affiliations:** Biochemistry and Molecular Biology Division, Agrochemistry and Biochemistry Department, Faculty of Sciences and Multidisciplinary Institute for Environmental Studies (IMEM), University of Alicante, Ap. 99, E-03080 Alicante, Spain; rosa.martinez@ua.es

**Keywords:** heterologous gene expression, homologous gene expression, recombinant proteins, metalloenzymes, denitrification, haloarchaea, *Haloferax* sp

## Abstract

Haloarchaea (halophilic microbes belonging to the Archaea domain) are microorganisms requiring mid or even high salt concentrations to be alive. The molecular machinery of these organisms is adapted to such conditions, which are stressful for most life forms. Among their molecular adaptations, halophilic proteins are characterized by their high content of acidic amino acids (Aspartate (Asp) and glumate (Glu)), being only stable in solutions containing high salt concentration (between 1 and 4 M total salt concentration). Recent knowledge about haloarchaeal peptides, proteins, and enzymes have revealed that many haloarchaeal species produce proteins of interest due to their potential applications in biotechnology-based industries. Although proteins of interest are usually overproduced in recombinant prokaryotic or eukaryotic expression systems, these procedures do not accurately work for halophilic proteins, mainly if such proteins contain metallocofactors in their structures. This work summarizes the main challenges of heterologous and homologous expression of enzymes from haloarchaea, paying special attention to the metalloenzymes involved in the pathway of denitrification (anaerobic reduction of nitrate to dinitrogen), a pathway with significant implications in wastewater treatment, climate change, and biosensor design.

## 1. Introduction

There is currently available a large variety of methods to create specific constructs for protein expression in a broad range of organisms. Moreover, restriction enzyme-free, ligation-independent, and recombinase-based cloning methods have enabled high-throughput protein expression for different purposes. These methods are also instrumental for modification of target genes, including gene truncations, site-specific mutagenesis, and domain swapping [1]. Therefore, thanks to the advances of these technologies and protocols, the homologous and heterologous expression of single genes or even complete operons has become reliable during the last few decades due to their potential applications, not only in basic but also in applied science. Gene overexpression is, in general, a step toward further studies about the structure and function of proteins, or it could be part of a biotechnological process to produce significant amounts of high-demand compounds, such as enzymes, hormones, etc. [2].

Traditionally, fungi, yeast, and bacteria have been used as hosts for the overexpression of gene coding for marketed proteins [3]. Since the early 1980s, *Escherichia coli* has probably been the most used bacteria for efficient expression of genes [4,5,6,7]. Over the years, research on heterologous gene expression using *E. coli* as the host has led to an improved capability to accumulate proteins in a soluble form, secrete proteins from the cell cytoplasm, accumulate proteins in the cytoplasmic membrane, and direct proteins to the outer membrane of the cell for surface display [8]. However, many heterologous polypeptides fail to fold into their native state when expressed in *E. coli*; instead, they are either degraded by the cellular proteolytic machinery or accumulated as protein aggregates referred to as inclusion bodies [9]. This is the case in heterologous expression of proteins from microorganisms showing a “rare” metabolism such as those from hyperthermophiles, halophiles, haloalkaliphiles, etc. The nature of these proteins (significant hydrophobic pattern, high content of amino acidic residues, requirements of high salt concentrations to promote protein folding and stability, requirements related to the activity of chaperons involved in their folding, etc.) makes difficult the production of soluble and active forms when using *E. coli* as the host for gene expression [10]. Some recent studies have combined different strategies to address this problem. The following approaches have contributed to the improvement of heterologous expression of proteins from extremophilic organisms: double promoter expression systems, development of new cloning methods, search for alternative hosts, or the design of processes to solubilize inclusion bodies and refold the proteins [11,12,13,14].

Haloarchaea are microbes belonging to the Archaea domain, characterized for having a high salt requirement to be alive. Literature data indicated that few proteins from haloarchaea can be successfully overproduced using *E. coli* [15,16]. In these studies, proteins obtained as inclusion bodies were solubilized in the presence of urea. In most cases, the proteins were then refolded by rapid dilution in high salt concentration buffers, thus recovering native structure and function [17,18,19]. Although the genetic manipulation of members of the Archaea domain is still difficult and scarce compared with bacteria, some recent studies have reported that homologous expression of haloarchaeal proteins is also possible (most of the work has been carried out with *Haloferax* species as the model organism) [20,21]. However, there are still limitations to overproduced haloarchaeal proteins containing metallocofactors by both heterologous and homologous approaches, mainly due to the imbalance between the three processes: apoprotein production, cofactors assembly and protein folding mediated by chaperons. The overexpression of genes encoding metalloenzymes involved in the nitrogen cycle in haloarchaea constitute a good example of such limitations. This review summarizes the main findings from works on heterologous and homologous expression of proteins from haloarchaea, as well as the main difficulties found when overexpressing metalloenzymes involved in denitrification in the group of organisms. Thus, this work continues to shed light on the main challenges to be analyzed in the future of this topic.

## 2. Heterologous Expression of Proteins from Haloarchaea

Although the development of viral vectors, systems for transformation, mutant production, and screening has permitted a significant increase of genetic manipulation of extremophilic microorganisms, the genetic manipulation of members of the Archaea domain is still difficult and limited compared with bacteria, as already mentioned before [22]. Archaea has been the focus of scientific attention during the last three decades, not only due to their biology but also due to the potential applications of the molecules that they produce (enzymes, bioplastics, carotenoids, antibiotics, etc.) [23,24,25]. Thus, enzymes from alkaliphiles, thermophiles, or halophilic archaea revealed as active and stable catalyzers at high-temperature and high salinity conditions, which are environments generally adverse to other enzymes, have potential applications in industry and biotechnology [26,27,28,29,30,31,32].

Halophilic enzymes are characterized by a relatively higher usage of acidic residues, a low frequency of lysine, and a high occurrence of amino acids with a low hydrophobic character. This composition makes the proteins’ surface acidic with a decrease in hydrophobic patches [33,34]. As an example, the analysis of *Haloferax mediterranei* glucose dehydrogenase structure showed an absence of very mobile side chains on the surface that allow the formation of a highly ordered multi-layered solvation shell. This feature is necessary under the water-limited conditions characterizing salty environments and industrial processes when looking for potential uses of these enzymes [34]. Halophilic enzymes show thermophilic character as well; consequently, they are stable in a broad range of temperatures, which make them attractive for the design of new industrial procedures [35,36]. They are also active and stable in media with low water activity as in the presence of organic solvents [37,38], even at low salt concentrations if they are encapsulated in reverse micelles. Under these conditions, halophilic enzymes could be used for biotechnological applications in nonaqueous media [39,40]. Many enzymes from haloarchaea with potential interest, such as dehydrogenases, glycosyl hydrolases, proteases, lipases, or esterases have been characterized at the laboratory scale, but no large-scale applications have been reported yet, mainly due to the difficulties of their overexpression by both homologous and heterologous processes.

The first approach to overexpress some of these haloarchaeal enzymes was heterologous expression using *E. coli* as the host. Table 1 summarizes some of the main works reported so far based on heterologous expression of haloarchaeal enzymes in bacteria.

**Figure 1:**
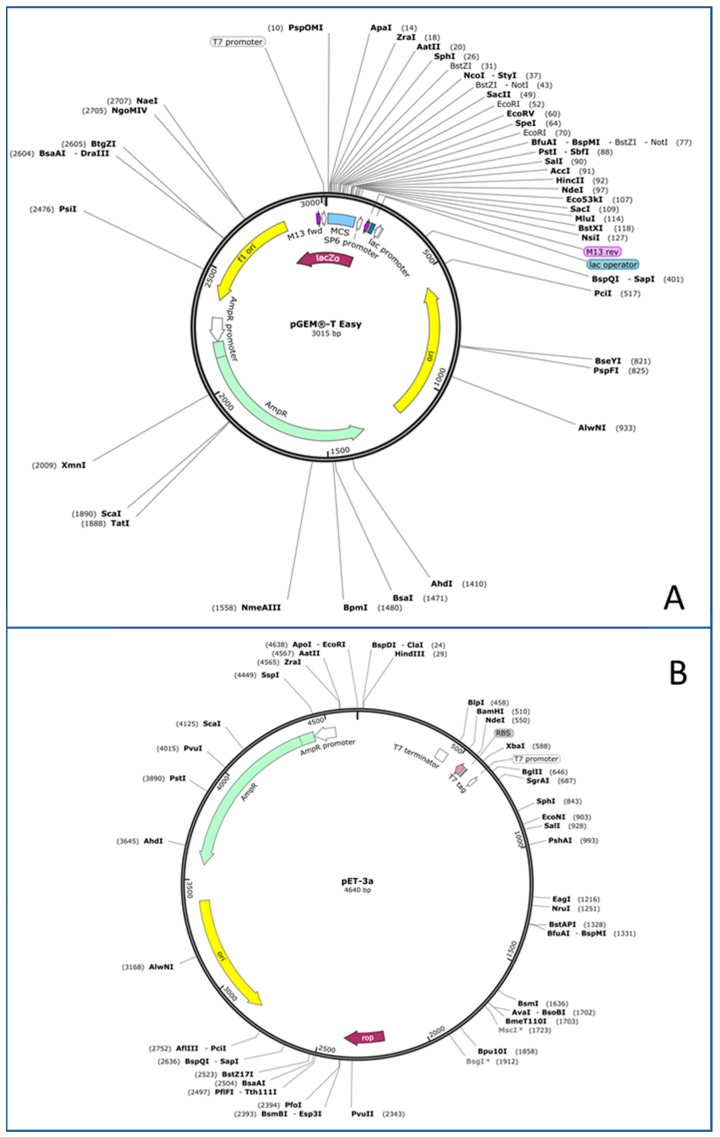
Main features of two of the most used vectors for protein expression in *E. coli.* Adapted from https://www.addgene.org/. (**A**) pGEM-T Easy plasmid. (**B**) PET-3a plasmid.

In the majority of these works, the enzymes have been expressed using different types of *E. coli* strains for cloning and protein expression (DH5a, BL21(DE3), XL-Blue, JM109, JM101 among others) and several cytoplasmic expression vectors such as pET (several versions: 3a, 3d, 11), pJAM, pGEM (several versions: pGEM-T easy vector, pGEM-7Zf, pGEM-4Z), or pET-Duet-1 (the last one for co-expression of proteins) (Figure 1).

The recombinant proteins were usually obtained as inclusion bodies, which were mainly solubilized in the presence of solvents showing chaotropic activity, such as buffers containing urea (up to 8 M). In most cases, the proteins were refolded by slow or rapid dilution in a high salt concentration buffer. The characterization of the recombinant proteins usually displayed activity and stability parameters closely related to those from the native proteins [17,18,44]. Thus, when the proteins are successfully obtained by this approach, the protocol show an important advantage: almost 100% of the proteins obtained as inclusion bodies are the recombinant proteins of interest; consequently, this process is simple, efficient, and yields enzymes of high purity in large amounts [19].

## 3. Homologous Expression of Proteins from Haloarchaea

Due to the limitations of genetic manipulation of members of the haloarchaea group, the homologous expression of proteins has been less explored than heterologous expression. However, several studies have confirmed that *Hfx. volcanii* can be easily manipulated, and consequently, it could be a good model organism as the host for the expression of haloarchaeal proteins at a mid or large scale. Table 2 summarizes most of the works reported on homologous expression of haloarchaeal enzymes. Homologous expression must be understood as the expression of proteins in the same species or in a closely related haloarchaea from a taxonomical point of view.

**Figure 2:**
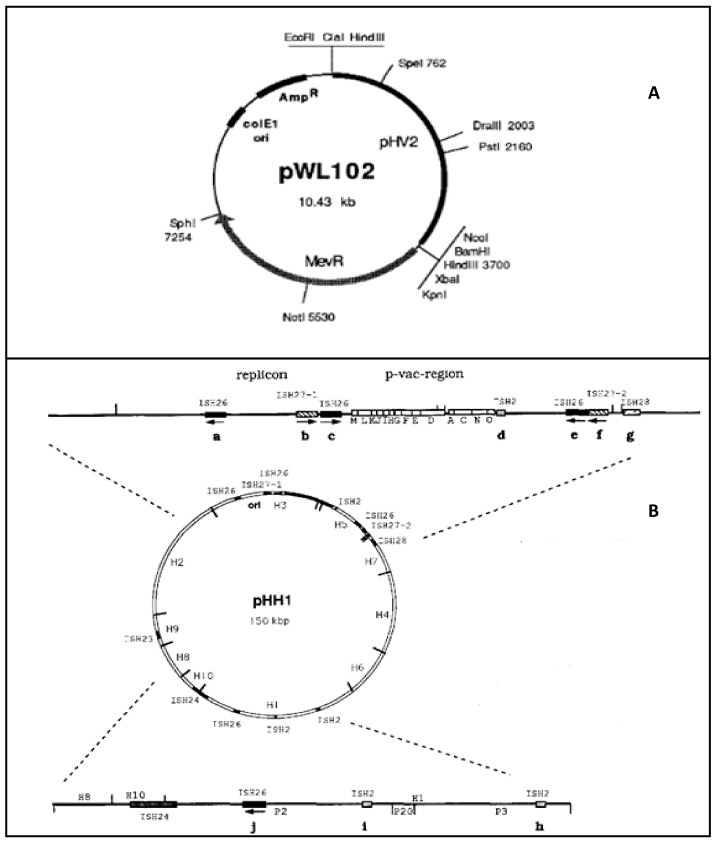
Main features of two vectors for homologous protein expression in haloarchaea. Adapted from 49 and 51. (**A**) PWL102, shuttle vector with demonstrated ability to transform *Haloferax volcanii*, *Haloferax mediterranei* (ATCC 33500), *Halobacterium halobium*, *Haloarcula hispanica* (ATCC 33960), and *Haloarcula vallismortis* (ATCC 29715) [49]. (**B**) pHH1 plasmid characterized by its high plasticity [56].

In the case of *Halobacterium halobium*, vector plasmids for its transformation were developed using the replicon region from the halobacterial phage H or from the plasmid pHH1 together with a DNA fragment conferring resistance to mevinolin. *H. halobium* P03 (a strain lacking pHH1 as well as the restriction endonuclease activity found in wild-type *H. halobium*), was used as the recipient strain. All *H. halobium* fragments tested for autonomous replication as well as the *Hfx. volcanii* vector pWL102 enabled stable plasmid maintenance in this strain [50,56] (Figure 2).

Regarding *Hfx. volcanii*, shuttle vectors were first described in the 1980s [57,58]. Currently, most of the protocols are based on cloning into a pGEM T-easy vector (Figure 1) in order to carry out the required enzymatic restrictions. Then, the gene of interest is usually inserted in a pJAS vector [21,59]. This halophilic vector contains a strong and constitutive ferredoxin promoter from *Halobacterium salinarum* and confers resistance to novobiocin. Strains such as *Hfx. volcanii* WFD11 are then transformed with the construction developed as described previously by Cline, among other authors [21,60]. It is interesting to highlight that *Hfx. volcanii* is currently the best haloarchaea to design and develop protocols to obtain recombinant proteins [61]. Moreover, it has been used as the host for bacterial protein expression. This is the case of pyruvate decarboxylase from *Zymomonas mobilis*, a Gram-negative, facultative anaerobic, nonsporulating, polarly flagellated, rod-shaped bacterium [62].

## 4. Expression of Metalloenzymes from Haloarchaea: Denitrification as a Case Study

**Figure 3:**
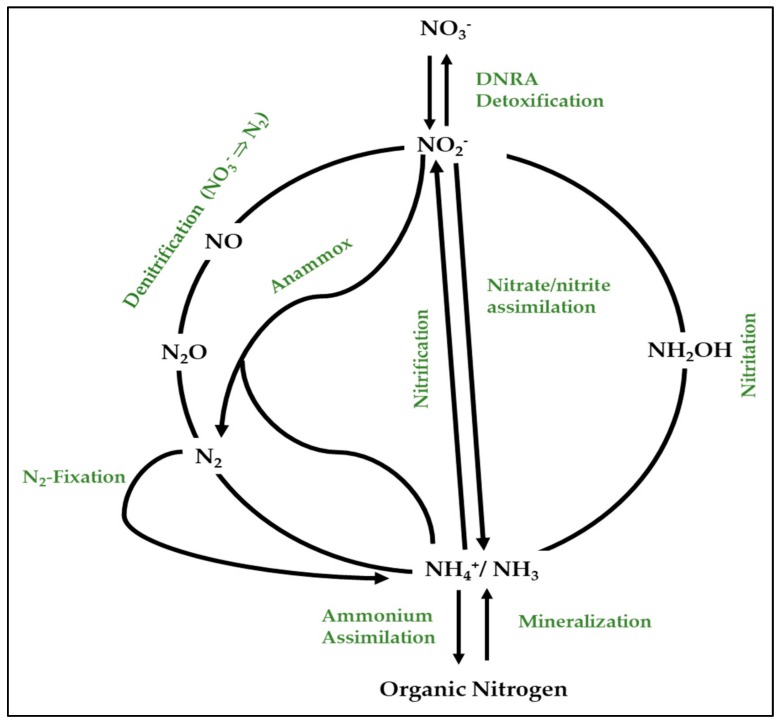
Major metabolic pathways of the biogeochemical nitrogen cycle. Different nitrogen compounds arise through the actions of several biological processes, the most prominent of which are termed nitrogen fixation, nitrification, dissimilatory nitrate reduction to ammonia (DNRA or nitrate ammonification), anaerobic ammonia oxidation (anammox), and denitrification (adapted from Thomson and co-workers) [71].

The nitrogen cycle (*N*-cycle) is one of the main biogeochemical cycles mainly driven by prokaryotes. It compromises different redox reactions sustaining assimilatory pathways or respiratory processes for energy conservation (Figure 3). Through this cycle, interconversions of nitrogen compounds are possible thanks to several enzymes, most of them being metalloenzymes [63,64]. In the case of denitrifying haloarchaea, nitrate reductases, for instance, contain iron–sulfur clusters and molybdenum cofactor (MoCo) [65,66]. Nitrite reductases contain siroheme and iron–sulfur clusters in the case of the assimilatory type or copper at the active site in the case of the respiratory type [42,67]. Other proteins involved in electron transfer in the pathways of the *N*-cycle such as Rieske proteins or cytochromes also contain hemes or iron–sulfur clusters of different types ([2Fe–2S], [3Fe–4S], [4Fe–4S]). Some of these metalloenzymes from bacteria and fungi have been successfully obtained as recombinant proteins in *E. coli* [68,69,70], which is not the case for the enzymes from haloarchaea.

**Figure 4:**
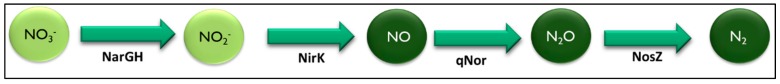
Summary of the reactions sustaining denitrification in haloarchaea, an anaerobic pathway in which nitrate is used as the final electron acceptor for respiratory purposes. The acronyms along the arrows refer to the names of each of the enzymes catalyzing these reactions.

Among the respiratory processes of the *N*-cycle, denitrification is one of the best studied anaerobic metabolic pathways in haloarchaea, particularly in members of the *Haloferax* genus (mainly in *Hfx. mediterranei*, *Hfx. volcanii*, and *Hfx. denitrificans*) [72] (Figure 4). In this pathway, nitrate (NO_3_^−^) is used by the cells as the final electron acceptor under anoxia. Then, NO_3_^−^ is further reduced to nitrite (NO_2_^−^) and gaseous products (nitric oxide (NO), nitrous oxide (N_2_O), and dinitrogen (N_2_)). Some denitrifiers are complete, i.e., nitrate is fully reduced to dinitrogen thanks to four key enzymes: respiratory nitrate reductase (Nar), respiratory nitrite reductase (Nir), nitric oxide reductase (Nor), and nitrous oxide reductase (Nos). However, the process is often incomplete (partial denitrification), leading to the release of the gaseous intermediates NO and N_2_O, which affect the environment [72,73,74].

As the *N*-cycle has important environmental implications, this biogeochemical cycle has become a major research topic worldwide during the last few years, especially with respect to its implications in climate change, global warming, and bioremediation [63]. The connections between the *N*-cycle and climate change are due to the release of these gases (NOx) to the atmosphere, which is harmful, because these gases are responsible for the destruction of the ozone layer and contribute to the greenhouse effect [72,73]. Regarding bioremediation, few metabolic pathways of the nitrogen cycle, such as denitrification or anammox, are useful and efficient for wastewater treatments [73,75]. Moreover, the enzymes of denitrification are of interest due to their potential uses in biotechnology: design of biosensors to measure nitrate or nitrite in wastewater, immobilization of enzymes for processes based on electrochemistry (wastewater treatments), etc. [75,76,77,78].

**Figure 5:**
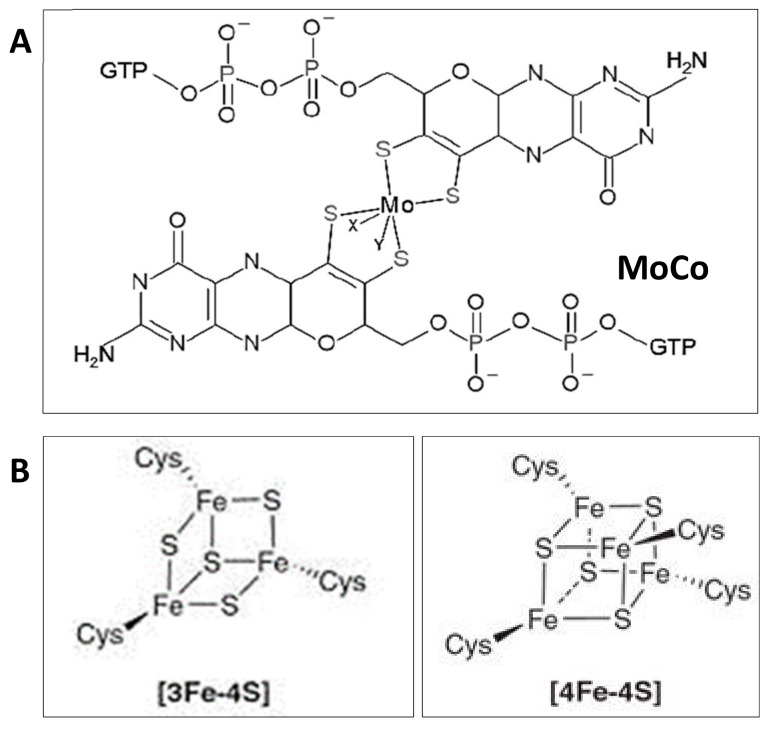
Structure of the main cofactors found in respiratory nitrate reductases in haloarchaea. (**A**) molybdenum cofactor (MoCo), which is located at the catalytic subunit of the enzyme [80] and (**B**) iron–sulfur clusters located in both catalytic and electron transfer subunits [65,79]. Molecules drawn with BioVIA Draw 2019 [81].

Three of these four enzymes are metalloenzymes in haloarchaea:

(i) NarGH (I): Respiratory nitrate reductase. It catalyzes the first reaction of denitrification in which NO_3_^−^ is reduced to NO_2_^−^. In general, the Nar complex in haloarchaea is a heterotrimer composed of a catalytic subunit (NarG) that binds a bismolybdopterin guanine dinucleotide (bis-MGD) cofactor for nitrate reduction and iron–sulfur clusters for electron transfer, an electron transfer subunit with four iron–sulfur centers (NarH), and a subunit similar to bacterial NarI, which is a di-b-haem integral membrane quinol dehydrogenase subunit [79] (Figure 5). This enzyme is usually a membrane-bound enzyme (facing the positive side of the membrane in the case of halophilic archaea) [79].
(ii) NirK: Respiratory nitrite reductase. It catalyzes the reduction of NO_2_^−^ to NO (gas). In haloarchaea, it is a Cu-type homodimeric enzyme belonging to the green Cu-NiR group [21,72].
(iii) NosZ: Nitrous oxide reductase. It catalyzes the reduction of N_2_O to N_2_ (last reaction of denitrification in the case of complete denitrifiers). At the time of writing this communication, none of the Nos from haloarchaea have been characterized from a biochemical point of view. From the analysis of the genes coding for it, it can be assumed that they are structurally complex Cu-containing enzymes similar to bacterial NosZ [72].

The first approach carried out to perform heterologous expression of respiratory nitrate reductase from *Haloferax mediterranei* using *E. coli* as the host was been successful. In this case, genes coding for the catalytic and the electron transfer subunit (NarG and NarH, respectively) were overexpressed and obtained as inclusion bodies. The inclusions bodies were solubilized in urea as previously described in the literature [19,44], and the refolding of the protein was performed by (i) rapid dilution or (ii) slow dilution with incubation at 4 °C or room temperature. High salt concentration buffers supplemented with sources of iron, sulfur, and molybdenum (at different concentrations between 0 and 1 mM) were used for the dilution. Other approaches already assayed in haloarchaea were based on the overexpression of genes belonging to the Nar gene cluster (it includes a chaperon and a protein involved in the biosynthesis of MoCo cofactor) [65,79], but positive results have not been reported yet. Regarding NirK from haloarchaea, the best results for its overproduction have been reported for the overexpression of the *nirK* gene from *Hfx. mediterranei* in *Hfx. volcanii* [21]. Although this is not homologous expression stricto sensu, *Hfx. volcanii* is revealed as a good host to carry out “homologous expression” of haloarchaeal genes in order to produce proteins of interest (mainly metalloprotein). Finally, no study has been reported to date on heterologous or homologous expression of a haloarchaeal nitrous oxide reductase.

Apart from the difficulties of heterologous or homologous expression of haloarchaeal enzymes involved in denitrification, another limitation to be highlighted is the purification of these proteins. Most of them are membrane proteins, and in those cases in which the enzymes have been characterized, it is possible to conclude that the TAT system is involved in the exportation of these membrane enzymes [64,72,73]. Some of the protocols successfully described to purify denitrifying enzymes from haloarchaeal membrane enzymes involve the use of detergents such as Triton X-10 [65]. Thus, purification must be carried out by using buffers with high salt concentration (to keep the native structure and activity) and detergents. The main characteristics of such types of buffers are their high density and viscosity, which negatively affect the chromatographic steps usually considered for protein purification [65,66,79]. Nevertheless, protocols for purification of respiratory nitrate reductases located in the membrane have been reported from *Haloferax mediterranei*, for instance [65], in which ionic exchange and hydrophobic chromatographies allow the highest purification fold, keeping constant a high value of specific enzymatic activity [65]. Optimization of both homologous protein overexpression and subsequent protein purification will promote potential uses of denitrifying enzymes in biotechnology and industries. Thus, those enzymes could be of high interest for the design and development of enzymatic immobilization procedures for wastewater treatments or the design of enzyme-based biosensors to monitor nitrate/nitrite concentrations in water containing high salt concentrations, which is one of the challenges to be overcame in the near future [76,77,78].

## 5. Conclusions

Although several protocols have been developed and refined to produce and purify some recombinant proteins from haloarchaea using mesophilic bacteria such as *E. coli* as the host, those protocols do not allow successful overproduction of the majority of haloarchaeal proteins, mainly those containing metallocofactors. The development of a series of plasmid vectors and host strains for conditional overexpression of halophilic proteins in the haloarchaeon *Hfx. volcanii* during the last decade offers new approaches for large-scale production of haloarchaeal proteins and enzymes that are of interest in industry and biotechnology [20,82,83]. Nevertheless, new challenges have to be overcome to achieve the following: (i) coupling the production of the apoproteins with the synthesis of cofactors in the case of metalloproteins; (ii) exploring whether or not the culture media for these purposes must be enriched with metals to sustain the production of metallocofactors; and (iii) if metal supplementation is required, optimizing the compositions of culture media to avoid metal precipitation due to the high ionic strength of the cultures used for the growth of haloarchaea. Thus, the development of plasmid vectors to co-express metalloproteins from haloarchaea and the genes coding for chaperons or enzymes related to the biosynthesis of cofactors (such as [Fe–S] or MoCo) is one of the main targets to be achieved in the future. In this context, the minichaperone-based protein fusion system recently described could be beneficial to achieve the overexpression of haloarchaeal proteins [84,85] Finally, because each protein is unique and due to the complex interactions among the reagents in experiments, it is mandatory to set up reaction conditions that would be optimal for each specific process to get recombinant proteins. Therefore, methods for the optimization of experimental conditions based on a one-factor-at-a-time approach should be replaced by a carefully selected small set of experiments characterized by their low cost and low time requirements. Available software packages would facilitate the choice of the design of the experiments in order to predict the effect of each factor and the effects of their interactions on a process sustaining the production of recombinant proteins [86]. Other approaches such as recombinant protein expression in biofilms [87] should be explored in the case of overexpression of proteins from haloarchaea.

## Figures and Tables

**Table 1:** Enzymes and proteins from haloarchaea overexpressed by heterologous approaches.

Enzyme	Haloarchaea Species	Host for Overexpression	Reference
Co-expression of the RadA recombinase with the RadB paralog	*Haloferax volcanii*	*Escherichia coli*	[41]
Cysteine desulfurase	*Haloferax volcanii*	*Escherichia coli*	[42]
D-2-hydroxyacid dehydrogenase	*Haloferax mediterranei*	*Escherichia coli*	[43]
Dihydrolipoamide dehydrogenase and citrate synthase	*Haloferax volcanii*	*Escherichia coli*	[16]
Glucose dehydrogenase	*Haloferax mediterranei*	*Escherichia coli*	[44]
Halolysin-like protease	*Natrialba magadii*	*Escherichia coli*	[45]
Laccase	*Haloferax volcanii*	*Escherichia coli*	[46]
Mn-containing superoxide dismutase	*Halobacterium halobium*	*Escherichia coli*	[47]
NADP-dependent isocitrate dehydrogenase	*Haloferax volcanii*	*Escherichia coli*	[17]
NADP-glutamate dehydrogenase	*Haloferax mediterranei*	*Escherichia coli*	[18]
Rhodopsins	*Haloarcula marismortui*	*Escherichia coli*	[48]

**Table 2:** Enzymes and proteins from haloarchaea overexpressed by homologous approaches.

Enzyme	Haloarchaeal Species	Host for Overexpression	Reference
Dihydrolipoamide dehydrogenase	*Haloferax volcanii*	*Haloferax volcanii*	[49]
Proteins involved in the synthesis of gas vesicles	*Halobacterium halobium*	*Halobacterium halobium*	[50]
Alkaline serine protease (halolysin)	unidentified halophilic archaea	*Haloferax volcanii*	[51]
Mn-containing superoxide dismutase (SOD)	*Halobacterium cutirubrum* and *Halobacterium volcanii*	*Halobacterium cutirubrum* and *Halobacterium volcanii*	[52]
Peptidases	*Haloferax volcanii*	*Haloferax volcanii*	[53]
Respiratory nitrite reductase (Cu-NirK)	*Haloferax mediterranei*	*Haloferax volcanii*	[21]
Alcohol dehydrogenases	*Haloferax volcanii*	*Haloferax volcanii*	[54]
β-galactosidase	*Halorubrum lacusprofundi*	*Halobacterium sp.* NRC-1	[55]
